# Walking naturally after spinal cord injury using a brain–spine interface

**DOI:** 10.1038/s41586-023-06094-5

**Published:** 2023-05-24

**Authors:** Henri Lorach, Andrea Galvez, Valeria Spagnolo, Felix Martel, Serpil Karakas, Nadine Intering, Molywan Vat, Olivier Faivre, Cathal Harte, Salif Komi, Jimmy Ravier, Thibault Collin, Laure Coquoz, Icare Sakr, Edeny Baaklini, Sergio Daniel Hernandez-Charpak, Gregory Dumont, Rik Buschman, Nicholas Buse, Tim Denison, Ilse van Nes, Leonie Asboth, Anne Watrin, Lucas Struber, Fabien Sauter-Starace, Lilia Langar, Vincent Auboiroux, Stefano Carda, Stephan Chabardes, Tetiana Aksenova, Robin Demesmaeker, Guillaume Charvet, Jocelyne Bloch, Grégoire Courtine

**Affiliations:** 1 NeuroX Institute, School of Life Sciences, Ecole Polytechnique Fédérale de Lausanne (EPFL), Geneva, Switzerland; 2 Department of Clinical Neuroscience, Lausanne University Hospital (CHUV) and University of Lausanne (UNIL), Lausanne, Switzerland; 3 NeuroRestore, Defitech Center for Interventional Neurotherapies, EPFL/CHUV/UNIL, Lausanne, Switzerland; 4 Univ. Grenoble Alpes, CEA, LETI, Clinatec, Grenoble, France; 5 Medtronic, Minneapolis, MN USA; 6 Department of Engineering Science, University of Oxford, Oxford, UK; 7 Department of Rehabilitation, Sint Maartenskliniek, Nijmegen, the Netherlands; 8 ONWARD Medical, Lausanne, Switzerland; 9 Univ. Grenoble Alpes, CHU Grenoble Alpes, Clinatec, Grenoble, France

**Keywords:** Brain-machine interface, Spinal cord diseases, Spinal cord

## Abstract

A spinal cord injury interrupts the communication between the brain and the region of the spinal cord that produces walking, leading to paralysis^1,2^. Here, we restored this communication with a digital bridge between the brain and spinal cord that enabled an individual with chronic tetraplegia to stand and walk naturally in community settings. This brain–spine interface (BSI) consists of fully implanted recording and stimulation systems that establish a direct link between cortical signals^3^ and the analogue modulation of epidural electrical stimulation targeting the spinal cord regions involved in the production of walking^4–6^. A highly reliable BSI is calibrated within a few minutes. This reliability has remained stable over one year, including during independent use at home. The participant reports that the BSI enables natural control over the movements of his legs to stand, walk, climb stairs and even traverse complex terrains. Moreover, neurorehabilitation supported by the BSI improved neurological recovery. The participant regained the ability to walk with crutches overground even when the BSI was switched off. This digital bridge establishes a framework to restore natural control of movement after paralysis.

## Abstract (web summary)

A reliable digital bridge restored communication between the brain and spinal cord and enabled natural walking in a participant with spinal cord injury.

## Main

To walk, the brain delivers executive commands to the neurons located in the lumbosacral spinal cord^7^. Although the majority of spinal cord injuries do not directly damage these neurons, the disruption of descending pathways interrupts the brain-derived commands that are necessary for these neurons to produce walking^8^. The consequence is permanent paralysis.

We previously showed that epidural electrical stimulation targeting the individual dorsal root entry zones of the lumbosacral spinal cord enables the modulation of specific leg motor pools^9–12^. In turn, recruiting these dorsal root entry zones with preprogrammed spatiotemporal sequences replicates the physiological activation of leg motor pools underlying standing and walking^4,5,11,13,14^. These stimulation sequences restored standing and basic walking in people with paralysis due to a spinal cord injury. However, this recovery required wearable motion sensors to detect motor intentions from residual movements or compensatory strategies to initiate the preprogrammed stimulation sequences^5^. Consequently, the control of walking was not perceived as completely natural. Moreover, the participants showed limited ability to adapt leg movements to changing terrain and volitional demands.

Here, we suggest that a digital bridge^13,15–19^ between the brain and spinal cord would enable volitional control over the timing and amplitude of muscle activity, restoring more natural and adaptive control of standing and walking in people with paralysis due to spinal cord injury.

## Digital bridge from brain to spinal cord

**Fig. 1:**
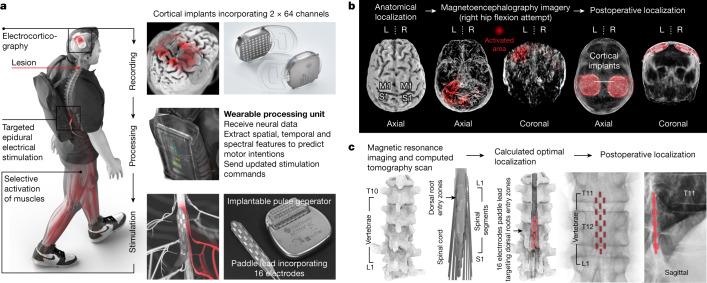
Design, technology and implantation of the BSI. **a**, Two cortical implants composed of 64 electrodes are positioned epidurally over the sensorimotor cortex to collect ECoG signals. A processing unit predicts motor intentions and translates these predictions into the modulation of epidural electrical stimulation programs targeting the dorsal root entry zones of the lumbosacral spinal cord. Stimulations are delivered by an implantable pulse generator connected to a 16-electrode paddle lead. **b**, Images reporting the pre-operative planning of cortical implant locations, and postoperative confirmation. L, left; R, right. **c**, Personalized computational model predicting the optimal localization of the paddle lead to target the dorsal root entry zones associated with lower limb muscles, and postoperative confirmation.

To establish this digital bridge, we integrated two fully implanted systems that enable recording of cortical activity and stimulation of the lumbosacral spinal cord wirelessly and in real time (Fig. 1a).

To monitor electrocorticographic (ECoG) signals from the sensorimotor cortex, we leveraged the WIMAGINE technology^3,20^. WIMAGINE implants consist of an 8-by-8 grid of 64 electrodes (4 mm × 4.5 mm pitch in anteroposterior and mediolateral axes, respectively) and recording electronics that are embedded within a 50 mm diameter, circular-shaped titanium case that has the same thickness as the skull. The geometry of the system favours close and stable contact between the electrodes and the dura mater, and renders the devices invisible once implanted within the skull.

**Extended Data Fig. 1:**
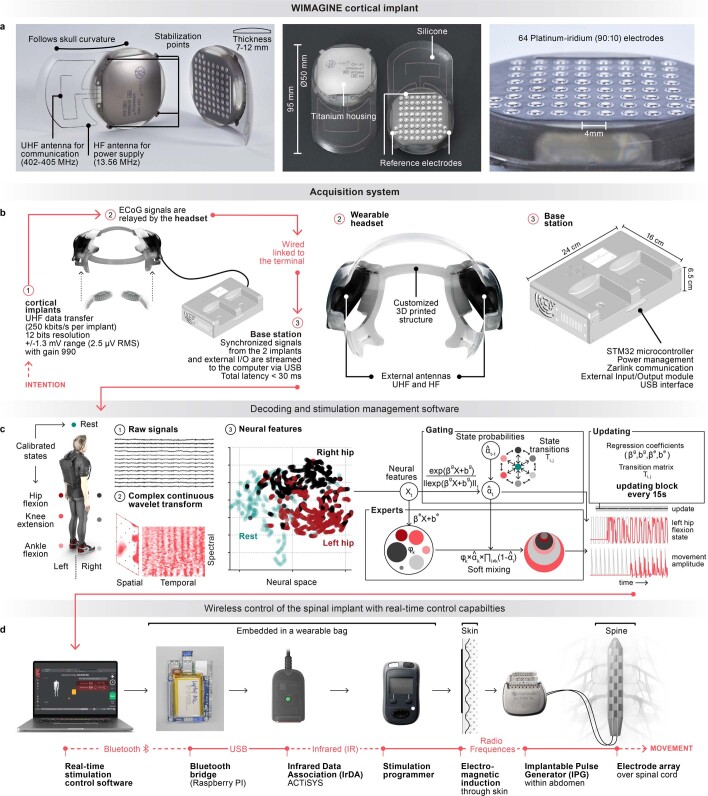
Technological and computational design underlying the *BSI*. **a**, Photographs reporting the geometry and features of the WIMAGINE implant, including 64 platinum-iridium (90:10) electrodes with 4 mm x 4.5 mm pitch (in antero-posterior and medio-lateral axes respectively). Two external antennas are embedded within the implant. The first antenna powers the implanted electronics through inductive coupling at high frequency (HF, 13.56 MHz) while the second ultrahigh frequency antenna (UHF, 402-405 MHz) transfers the recorded signals outside the body. **b**, Two external antennas embedded in a personalized 3D-printed headset power the implant and recover the streamed signals that are then transferred to a base station. This base station manages the powering of the implants, synchronization and conditioning of the raw data. **c**, A decoding pipeline computes temporal, spectral and spatial features embedded in the ECoG signals related to the intention to move. These features are then uploaded into the decoding algorithm that decodes the attempts to move the lower limbs based on a tailored, recursive exponentially weighted Markov-switching multi-linear model algorithm^26^. This algorithm is a mixture of multilinear experts’ algorithm integrating a Hidden Markov Model (HMM) classifier, called ***gating***, and a set of independent regression models, called ***experts***. The ***gating*** classifier predicts the joint that is intended to be mobilized (i.e. hip, knee or ankle on each side) as well as resting state, while each ***expert*** is dedicated to predicting the direction and relative amplitude of the intended movement. When updating is allowed, every 15 s, the coefficients of both linear regressions (β^gate^, b^gate^, β^expert^, b^expert^) are updated through recursive partial least square along with the coefficients of the transition matrix T corresponding to the number of transitions between each states during this 15s period (i.e. 150 new transitions). To support the production of standing and walking, the outputs of the model are encoded into updates of joint-specific stimulation programs that are constrained within pre-established functional ranges of amplitudes. **d**, A tailored, medical-grade software sends these updates to the implanted pulse generator through a chain of wireless communication systems, eventually delivering the stimulation through a paddle array implanted epidurally over the lumbosacral spinal cord.

Two external antennas are embedded within a personalized headset that ensures reliable coupling with the implants. The first antenna powers the implanted electronics through inductive coupling (high frequency, 13.56 MHz), whereas the second, ultrahigh frequency antenna (UHF, 402–405 MHz) transfers ECoG signals in real time to a portable base station and processing unit, which generates online predictions of motor intentions on the basis of these signals (Extended Data Fig. 1).

The decoded motor intentions are then converted into stimulation commands that are transferred to tailored software running on the same processing unit.

These commands are delivered to the ACTIVA RC implantable pulse generator (Fig. 1a), which is commonly used to deliver deep brain stimulation in patients with Parkinson’s disease. We upgraded this implant with wireless communication modules that enabled real-time adjustment over the location and timing of epidural electrical stimulation with a latency of about 100 ms (Extended Data Fig. 1).

Electrical currents are then delivered to the targeted dorsal root entry zones using the Specify 5-6-5 implantable paddle lead, which consists of an array incorporating 16 electrodes.

This integrated chain of hardware and software established a wireless digital bridge between the brain and the spinal cord: a brain–spine interface (BSI) that converts cortical activity into the analogue modulation of epidural electrical stimulation programs to tune lower limb muscle activation, and thus regain standing and walking after paralysis due to a spinal cord injury (Supplementary Video 1).

## Neurosurgical implantation of the BSI

In the context of the Stimulation Movement Overground (STIMO)-BSI clinical trial (clinicaltrials.gov, NCT04632290), we enroled a 38-year-old male who had sustained an incomplete cervical (C5/C6) spinal cord injury during a biking accident ten years before. He had previously participated in the STIMO clinical trial (clinicaltrials.gov, NCT02936453), which involved a five-month neurorehabilitation programme supported by targeted epidural electrical stimulation of the spinal cord^4,5^. This programme enabled him to regain the ability to step with the help of a front-wheel walker. Despite continued use of the stimulation at home, for approximately three years, he had reached a neurological recovery plateau, which motivated him to enrol in STIMO-BSI.

To guide the implantation of the BSI, we developed pre-operative planning procedures that enabled us to optimize the positioning of the recording and stimulation implants over the brain and spinal cord.

The BSI requires the detection of neural features related to the intention to move the left and right lower limbs. To identify the cortical regions most responsive to the attempt to move each joint of the lower limbs, we acquired anatomical and functional imaging data based on computerized tomography and magnetoencephalography (Fig. 1b). These acquisitions identified the regions of the cerebral cortex that responded more robustly to the intention to move the left and right lower limbs. We integrated this information with anatomical constraints, to define the optimal positioning of the two ECoG recording implants that aim to decode left and right lower limb movements. The location of both implants was uploaded onto a neuronavigation system to establish the pre-operative planning of the neurosurgical intervention.

Under general anaesthesia, a bicoronal incision of the scalp was performed to enable two circular-shaped craniotomies over the planned locations of the left and right hemispheres, using a tailor-made circular trephine that matched the diameter of the implants. We then replaced the bone flaps with the two implantable recording devices, before closing the scalp.

The paddle lead was positioned over the dorsal root entry zones of the lumbar spinal cord during the STIMO clinical trial. The optimal position of the lead was identified using a personalized model of the spine elaborated from high-resolution structural imaging^5^ (Fig. 1c). The final location was optimized intra-operatively on the basis of electrophysiological recordings^4,5^. The implantable pulse generator, which was connected to the lead, was inserted into an abdominal subcutaneous pocket.

The participant was discharged 24 h after each neurosurgical intervention.

## Setup of cortical and spinal implants

The calibration of the BSI required two independent procedures to select the features of ECoG recordings that discriminate the intention to move, and to configure stimulation programs that modulate specific ensembles of lower limb muscles.

**Fig. 2:**
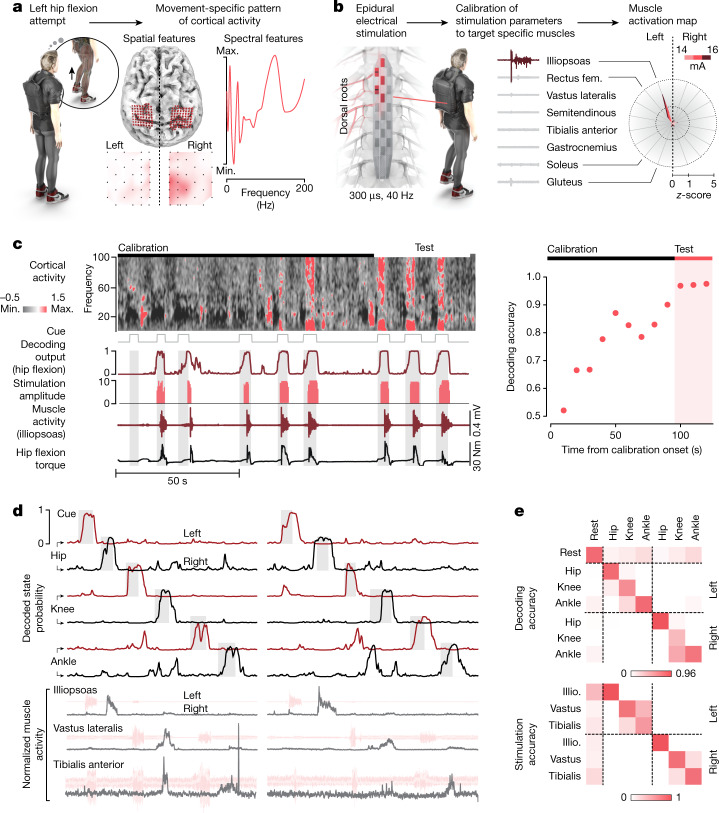
Calibration of the BSI. **a**, Identification of the spatial and spectral distributions of ECoG feature weights related to attempted left hip flexions. **b**, Calibration of anode/cathode configurations and stimulation parameters (frequency, range of amplitudes) to elicit left hip flexions, including electromyographic signals from lower limb muscles. The polar plot reports the relative amplitude of muscle responses for the optimal configuration to target left hip flexors over the range of functional stimulation amplitudes (300 µs, 40 Hz, 14–16 mA). **c**, Online calibration of the BSI to enable volitional hip flexion in a seated position. Representative sequence reporting spectrogram, decoding probability and proportional modulation of stimulation amplitudes together with the resulting muscle activity and torque. The plot reports the convergence of the model over time, reaching 97 ± 0.4% after 90 s. **d**, Similar representations after the calibration of the BSI to enable the control over hip, knee and ankle joints of the lower limbs. **e**, Confusion matrices reporting the decoding accuracy for each joint (74 ± 7% s.e.m.) and the accuracy of the stimulation for each targeted muscle group (83 ±  6% s.e.m.).

**Extended Data Fig. 2:**
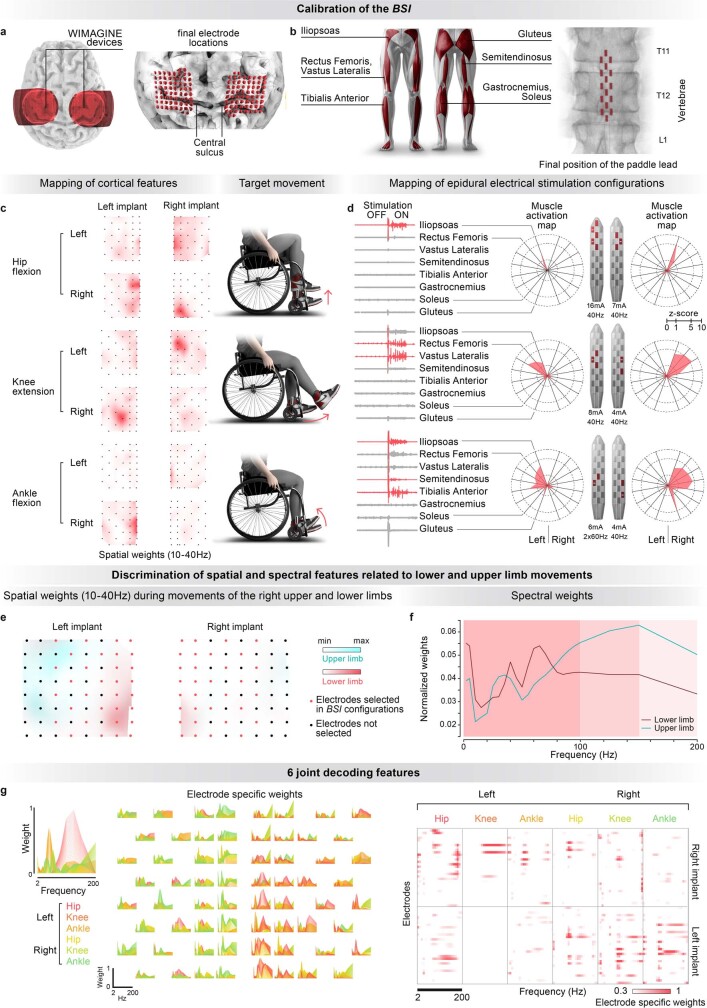
Calibration of the *BSI*. **a**, Post-operative localization of the cortical implants over the segmented brain of the participant that confirms the appropriate positioning of the 64-electrode grids over the activated regions of the primary motor cortex responding to attempted lower limb movements, as measured during functional magnetoencephalographic recordings. **b**, Post-operative localization of the paddle lead over the lumbosacral spinal cord to target lower limb muscles. **c**, Projection of linear regression weights associated with different lower limb movements (depicted on body schemes) onto the location of the implants, revealing the spatial segregation of movement-specific features. **d**, Electromyographic activity recorded from several lower limb muscles following a burst of epidural electrical stimulation using the more selective electrode configurations (schemes) and parameters (reported) translated into polar plots reporting the amplitude of muscle responses. **e**, Spatial distribution of linear regression weights associated with upper versus lower limb movements over the grid of 64 electrodes from each cortical implant. The firmware enabled the selection of 32 electrodes within the 64 electrodes of each implant. The red dots indicate the 32 selected electrodes from each implant based on the amount of identified movement-related information for each of the 64 electrodes. **f**, Spectral distribution of linear regression weights associated with upper versus lower limb movements, highlighting the importance of high sampling density in low frequencies compared to high frequencies. This ensemble of features guided the parameterization of the decoders. **g**, Detailed representation of the spatial and spectral repartition of weights associated with decoding of the 6 different lower limb joint movements.

The first procedure consisted of extracting the spatial, spectral and temporal features of ECoG signals that were linked to the intention to mobilize each joint of both lower limbs. For this purpose, the participant was asked to attempt hip, knee and ankle movements of the left and right sides in a seated position, during which ECoG signals were recorded concurrently. This mapping enabled the identification of the electrodes, spectral features and temporal windows that captured the larger amount of movement-related information^4,21–24^ (Fig. 2a and Extended Data Fig. 2). The electrodes that measured neural signals correlating with leg movements were located on the most medial aspect of the implant, rostral to the central sulcus, as expected on the basis of pre-operative magnetoencephalographic recordings. The spatial distribution of these electrodes followed a somatotopy that enabled the accurate discrimination of hip, knee and ankle movements (Extended Data Fig. 2c). On the other hand, upper limb-related movements coincided with the modulation of ECoG signals measured through electrodes located on the lateral aspect of the implant (Extended Data Fig. 2f). Movement-related information was contained over the entire range of beta and gamma frequency bands of the ECoG signals (Fig. 2a and Extended Data Fig. 2g). This procedure enabled us to configure the implants with optimal features to enable the participant to operate the BSI (Extended Data Fig. 2f,g).

The second procedure consisted of configuring stimulation programs (Fig. 2b). Epidural electrical stimulation of the spinal cord can modulate specific ensembles of motor pools through the recruitment of the dorsal root entry zones projecting to the spinal cord regions wherein these motor pools reside^9,25^. In turn, optimized configurations of anodes and cathodes can steer electric fields towards specific subsets of dorsal root entry zones to modulate well-defined ensembles of motor neuron pools^5,9,25^. This physiological principle enables the regulation of extension and flexion movements from each joint. We leveraged this principle to configure a library of targeted epidural electrical stimulation programs that mobilized the hip, knee and ankle joints from both sides. Concretely, we configured combinations of anodes and cathodes, stimulation frequencies and amplitudes to steer electrical currents to achieve gradual control over the activity of the targeted muscle groups (Extended Data Fig. 2b–d).

## Adaptive online calibration of the BSI

We then leveraged the configurations of cortical and spinal implants to calibrate the BSI on the basis of a recursive, exponentially weighted Aksenova/Markov-switching multilinear algorithm that linked ECoG signals to the control of epidural electrical stimulation parameters (Extended Data Fig. 1).

The algorithm was designed to generate two separate predictions. First, a gating model calculated the probability of the intention to move a specific joint. Second, an independent multilinear model predicted the amplitude and direction of the intended movement. The adaptive properties of the algorithm enabled online, incremental parametrization of the models throughout the period of calibration. A hidden Markov model ensured the stability and robustness of the predictions^26^.

We then translated the predictions of the algorithm into an analogue controller that adjusted the amplitude of joint-specific stimulation commands. These updated commands were delivered to the implantable pulse generator every 300 ms.

As early as the first session after neurosurgical intervention, the algorithm calibrated a BSI that enabled the participant to control the relative flexion of the left and right hips of an avatar projected on a screen (Supplementary Video 2). We then integrated the analogue control over the stimulation amplitude to the algorithm. From a lying position, within less than two minutes the participant was able to control the activity of hip muscles to generate a torque with an accuracy of 97% (Fig. 2c).

We then expanded this BSI framework to enable the participant to control the relative amplitude of hip, knee and ankle joints bilaterally along with the resting state, amounting to a total of seven states. Using this proportional BSI combining seven states, the participant achieved gradual control over the movement of each joint bilaterally with an accuracy of 74 ± 7%, whereas the chance level was limited to 14% (Fig. 2d,e). The latency of the decoder was as low as 1.1 s (±0.15 s s.e.m.) for the seven states.

These early sessions validated the procedure for the rapid, robust and accurate calibration of a BSI operating over multiple dimensions.

## Immediate recovery of natural walking

We next asked whether this procedure supports the calibration of a BSI that restores natural control of walking.

Walking involves well-defined sequences of muscle activation patterns that support weight acceptance, propulsion and swing of the left and right lower limbs. These sequences coincide with the activation of motor pools located within well-segregated regions of the lumbosacral spinal cord^4,27^. Therefore, we selected the stimulation programs within the library that targeted muscles associated with weight acceptance, propulsion and swing functions, and linked these programs to decoding probabilities. We calibrated the BSI to enable the participant to control the relative amplitude of stimulation programs for weight acceptance and swing functions.

**Fig. 3:**
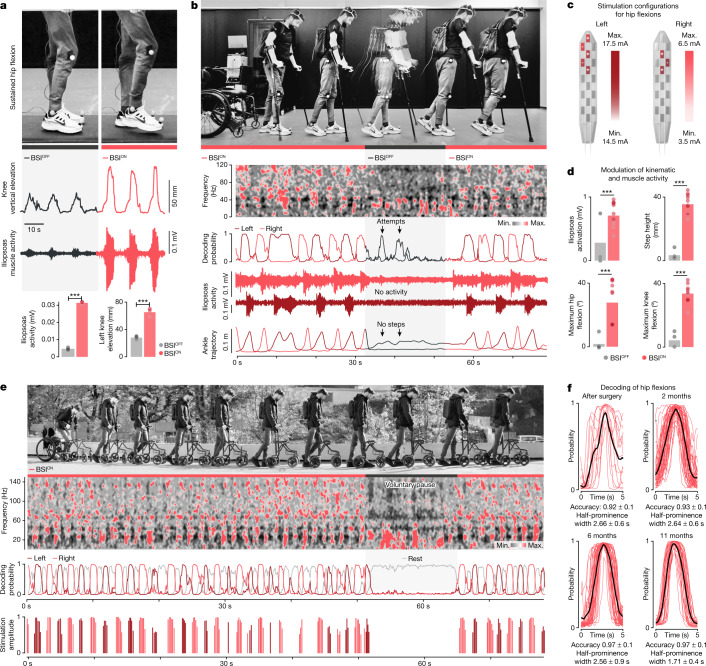
The BSI restores natural control of walking. **a**, Attempts to perform voluntary hip flexions without and with the BSI, including photographs, vertical elevation of the knee and hip flexor muscle activity. Bar plots report the mean values for these measurements. (*n* = 3 attempts per condition, unpaired one-tailed *t*-test, ***P < 0.001.) **b**, Chronophotography during walking with the BSI turned on, off and then on again. Note the two decoded attempts that do not lead to muscle activity nor the execution of steps. **c**, Range of stimulation amplitude during walking. **d**, Bar plots reporting mean values of kinematic and muscle activity parameters during walking with the BSI turned off and on, (*n* = 3 and 8 attempts for BSI^OFF^ and BSI^ON^, respectively, unpaired one-tailed *t*-test, ***P < 0.001, *P*(iliopsoas activation) = 3.4 × 10^−4^, *P*(step height) = 5.1 × 10^−10^, *P*(hip angle) = 2.7 × 10^−5^, *P*(knee angle) = 1.6 × 10^−9^). **e**, Chronophotography of standing (voluntary pause) and walking with the BSI outdoors. The spectrogram, probabilities of left and right steps and modulation of stimulation amplitudes illustrate the robustness of the performance and absence of false-positive detections during the voluntary pause. **f**, Plots report the probability of right hip flexions over consecutive steps measured during the first session after the neurosurgical implantation (*n* = 13 steps, accuracy = 0.92 ± 0.1 s.d., *w* = 2.66 ± 0.6 s s.d.), and at 2 (*n* = 46 steps, accuracy = 0.93 ± 0.1 s.d., *w* = 2.64 ± 0.6 s s.d.), 6 (*n* = 41 steps, accuracy = 0.97 ± 0.1 s.d., *w* = 2.56 ± 0.9 s s.d.) and 11 months (*n* = 29 steps, accuracy = 0.97 ± 0.1 s.d., *w* = 1.71 ± 0.4 s s.d.) after the first activation of the BSI using updated models (Extended Data Fig. 5).

We first tested this BSI during voluntary elevations of the foot while standing. After only 5 min of calibration, the BSI supported continuous control over the activity of hip flexor muscles, which enabled the participant to achieve a fivefold increase in muscle activity compared to attempts without the BSI (Fig. 3a).

**Extended Data Fig. 3:**
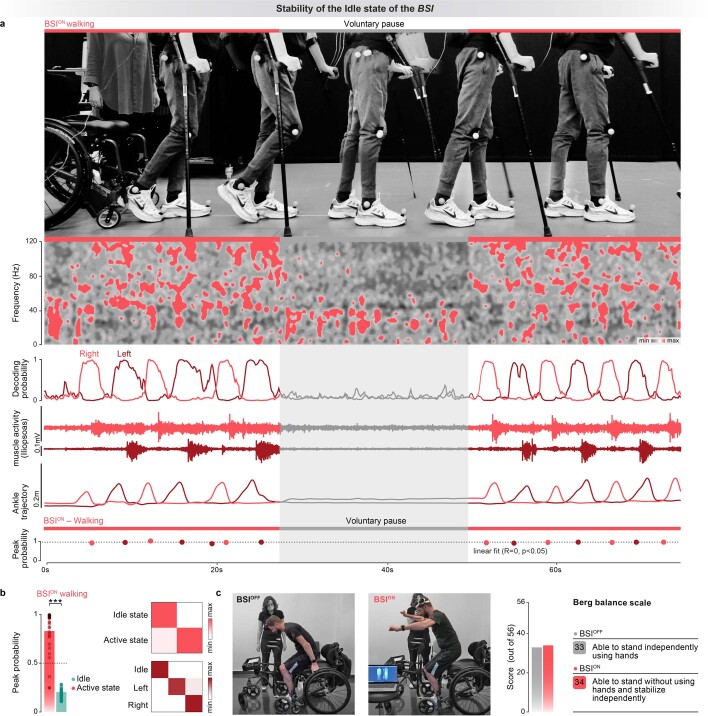
Stability of the decoder enables safe utilization of *BSI*. **a**, Chronophotography and associated spectrogram, probabilities of left and right steps, modulation of muscle activity, ankle height, and peak probability of step cycles during a sequence involving walking, a voluntary pause (30 s, instructed), and resuming walking. The absence of false positive detections illustrates the robustness of the *BSI*. **b**, The bar plot reports the peak probability of walk (active) versus idle state, together with the confusion matrices reporting the detected rest versus left and right swing states (n = 31 and n = 49 samples for idle and active states respectively, unpaired one-tailed t-test ***, P < 0.001). **c**, Photographs illustrating sit to stand capacities without and with the *BSI*, including bar plots reporting balance capacities (scores) measured using the Berg Balance Scale.

We provided the same configuration to support walking with crutches. The BSI enabled continuous, intuitive and robust control of walking (Fig. 3b). When the BSI was turned off, the participant instantly lost the ability to perform any step, despite detected attempts to walk from the modulation of cortical activity. Walking resumed as soon as the BSI was turned back on. The participant was able to decide whether to initiate stepping, walk continuously, stop or stand quietly without the detection of false-positives that would impair standing performance (Extended Data Fig. 3). Indeed, Berg Balance Scale assessments revealed that the BSI did not impair, and even slightly improved, overall balance abilities (Extended Data Fig. 3c).

**Extended Data Fig. 4:**
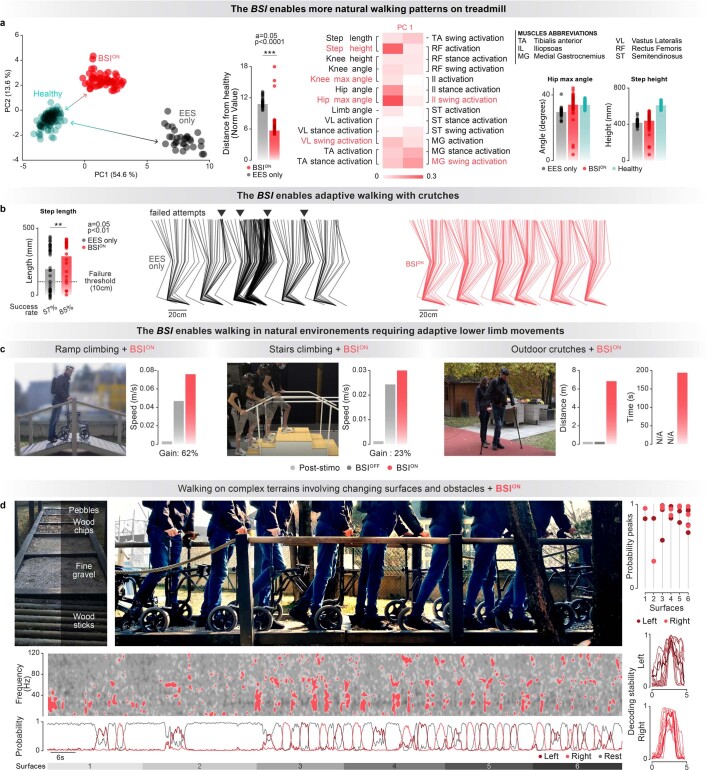
The *BSI* normalizes gait parameters and supports walking on complex terrains. **a**, Principal component (PC) analysis applied on kinematic and muscle activity parameters during walking on a treadmill with stimulation alone versus *BSI*. During stimulation alone conditions, a closed-loop controller based on motion sensors attached to the lower limbs determine the parameters of stimulation. Each dot represents a gait cycle. The bar plot reports the Euclidean distance in the PC space between each sample and the centroid of the healthy steps. (n = 119, n = 30 and n = 61 steps for healthy, EES only and *BSI* respectively, unpaired one-tailed t-test ***, P < 0.001). Compared to stimulation alone, the *BSI* enabled walking with gait features that were closer to those quantified in healthy individuals. This similitude is highlighted in the bar plots, which report the mean values of kinematic parameters with a high factor loading on PC1. **b**, Quantitative measure of step length while walking with crutches. Steps below 10 cm are considered failed as illustrated in the stick plot diagram. EES only condition showed significantly shorter step length due to increase of failed steps (n = 26, n = 43 for EES only and *BSI* respectively, Mann-Whitney U test one-tailed t-test **, P < 0.01). **c**, Photographs illustrating walking capacities, together with bar plots that report quantifications of performance during the execution of various walking paradigms, including walking up and down a ramp, climbing stairs, and walking with crutches overground. **d)** Walking on changing terrains with obstacles and different textures (6 surfaces), as illustrated in the scheme on the left. Conventions are the same as in previous figures. Decoding stability is shown by overlayed probability curves of right hip flexions over consecutive steps (n = 13 steps, Left accuracy = 0.89 +/− 0.1 std, w = 2.06 s +/− 0.6 s std), and Left accuracy (n = 13 steps, accuracy = 0.91 +/− 0.1 std, w = 2.06 s +/− 0.4 s std).

The participant reported that the BSI enabled a natural control over his movements during walking (Supplementary Video 2). We aimed to capture this subjective perception with quantified outcomes. For this purpose, we applied a principal component analysis to whole-body kinematics and muscle activity collected during walking on a treadmill with the BSI or with the same stimulation programs controlled in a closed loop on the basis of motion sensors attached to the feet. Compared to stimulation alone, the BSI enabled walking with gait features that were markedly closer to those quantified in healthy individuals (Extended Data Fig. 4a). The BSI ensured a continuous link between the intended movement and the modulation of stimulation protocols, which translated into the ability to walk overground independently with crutches. When the intended movements were detected from the motion sensors, the participant reported a frequent temporal mismatch between the detections and his intentions, which impaired his ability to walk under these conditions (Extended Data Fig. 4b).

## Navigation over complex terrain

We next aimed to investigate whether the BSI could enable an intuitive and natural control over complex activities of daily living that were not possible without the BSI.

When the participant enroled in STIMO, seven years after his accident, he was not able to walk independently. Completion of this clinical trial enabled him to regain basic walking when stimulation was turned on, albeit that this recovery required compensatory strategies to trigger the sequences of stimulation based on heel elevations. He also recovered partial mobility without stimulation. However, he experienced difficulties transitioning from standing to walking and stopping, and could only walk over flat surfaces. Moreover, he was not able to adjust lower limb movements to progress over ramps, overcome obstacles or climb up staircases—as is necessary to support mobility in everyday life.

To demonstrate that the BSI remedied these limitations, we designed a succession of models that emulated the conditions underlying these activities of daily living.

We first asked whether the participant was able to walk on steep terrain requiring adaptive modulation of the amplitude of muscle activity. With the BSI, the participant climbed up and down a steep ramp with ease, performing this task two times faster than without stimulation. The BSI also enabled high step clearance, as necessary to climb over a succession of stairs, negotiate obstacles and traverse changing terrains (Extended Data Fig. 4c,d). All these tasks were performed with the same BSI configuration, which proved highly reliable to support a broad variety of tasks with widely different constraints (Extended Data Fig. 4c,d).

## Long-term stability of the BSI

We next sought to assess the stability of the BSI. For this purpose, we quantified the stability of cortical signals and decoders over time, and the need to adjust stimulation programs.

**Extended Data Fig. 5:**
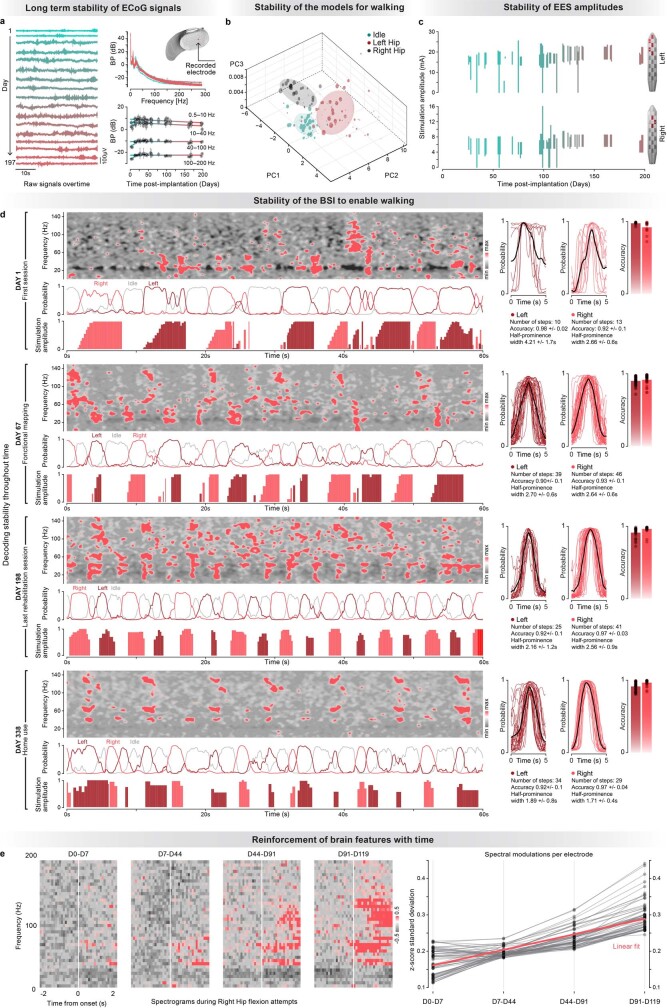
Long term stability of the *BSI* supporting walking. **a**, Recordings of the resting state were acquired regularly to evaluate the evolution of signal quality over time. Raw traces and power spectrum of an ECoG signal measured from a selected electrode are shown to illustrate the stability of the recorded signals. The plot reports the mean values of the power spectrum quantified over 2 min of resting state recorded at regular intervals over a period of nearly one year, showing a steady yet negligible decrease in signal quality over time (−0.03 dB/day). **b**, Plots reporting principal component analysis of gating coefficients from all the models used for supporting walking over the entire duration of the study. The size of each data point captures the relative time during which each model was used. **c**, Plots reporting the range of stimulation amplitudes and frequencies used over the entire course of the neurorehabilitation program, highlighting the robustness of the *BSI* over nearly six months of use. **d**, Spectrograms and decoding performance together with modulation of stimulation amplitude (relative) during self-paced walking enabled by the *BSI*. Plots report the probability of left and right hip flexion events (swing) measured over consecutive steps, and repeated at regular intervals over the entire time course of the clinical trial. **e**, Median spectrograms around the right hip flexion attempts during different time periods along training (n = 100 attempts in each period). The average rectified modulations show a significant increase with time (n = 64 electrodes, R2 = 0.68, P < 0.001).

**Extended Data Fig. 6:**
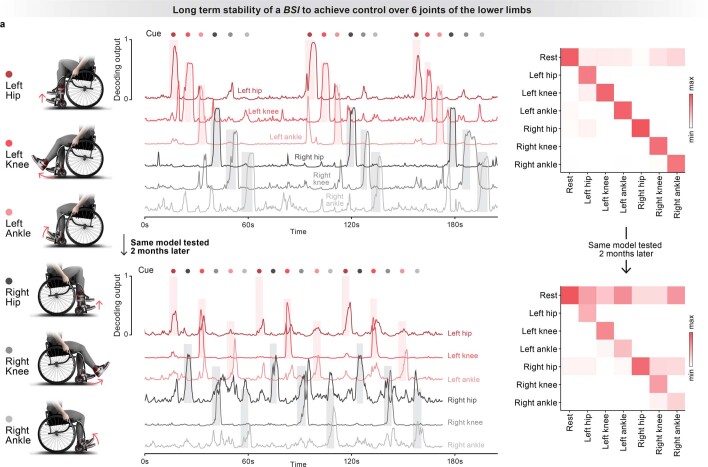
*BSI* supporting control over isolated lower limb movements. The same model was used to enable the participant to exert control over 6 joints from both sides during two sessions apart from 2 months. Conventions are the same as in previous figures.

After a transitory one-month period, during which cortical signals exhibited modest changes in the spectral content of the different frequency bands, ECoG signals remained stable over the following months (Extended Data Fig. 5a). The decrease in the spectral power was limited to 0.03 dB per day on average. This stability enabled robust performance. For example, we found that the same decoder enabled the participant to achieve gradual control over six joints despite a two-month interval between both sessions (Extended Data Fig. 6). We leveraged this robustness during neurorehabilitation, as we only recalibrated the BSI when deemed necessary by the participant and/or physiotherapists to promote the best possible functional performance. Despite these recalibrations, the features of the decoders remained remarkably stable over time (Extended Data Fig. 5b). Indeed, signal quality and decoding accuracy during walking has remained globally unchanged over nearly one year of use (Fig. 3f and Extended Data Fig. 5d). Whereas cortical features remained stable over time, we detected a progressive reinforcement of their modulation depth, which revealed gradual improvements in the ability of the participant to modulate his cortical activity when operating the BSI (Extended Data Fig. 5e).

The library of stimulation programs showed the same stability. The optimal range of stimulation amplitudes was dependent on the specific configuration of electrodes and targeted muscles (Extended Data Fig. 5c). However, these ranges of stimulation amplitudes have remained stable over one year of use, and stimulation thresholds did not change over time.

## Neurological recovery

**Fig. 4:**
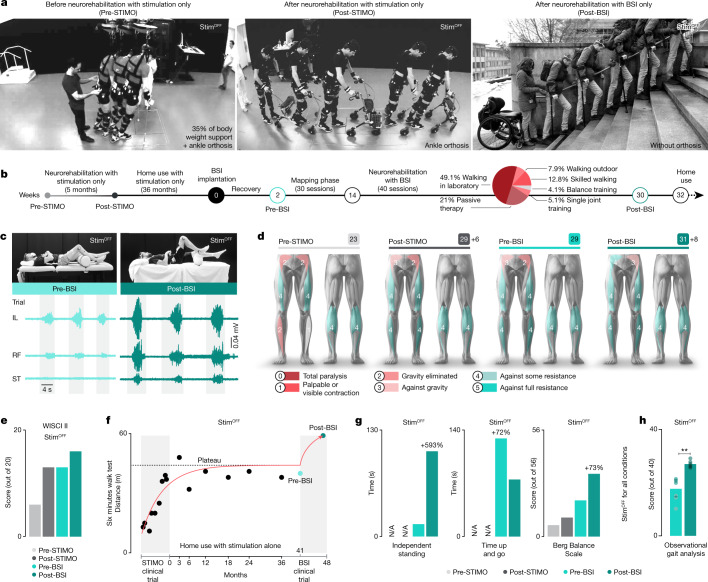
Neurological improvements following neurorehabilitation supported by the BSI in the absence of stimulation. **a**, Chronophotography illustrating the walking ability of the participant without any stimulation before enroling in the STIMO clinical trial (pre-STIMO), after its completion (post-STIMO) and after completion of the STIMO-BSI clinical trial (post-BSI). **b**, Timeline of the two clinical trials, including a pie chart reporting the time during which the various types of neurorehabilitation exercises were practised, as well as the home use of the BSI. **c**, Photographs showing the maximal hip flexion and associated flexor muscle activity before and after neurorehabilitation. **d**, Changes in lower limb motor scores over the course of both clinical trials. **e**, Plots reporting improvements in WISCI II scores over the course of both clinical trials. Neurorehabilitation supported by the BSI restored the capacity to walk over 10 m with crutches without any assistance or stimulation. **f**–**h**, Plots reporting quantifications of the 6 min walk test (**f**), weight-bearing capacity, time up and go, Berg Balance Scale (**g**) and observational gait analysis (**h**) (each dot refers to scores from a physiotherapist (*n* = 6, paired one-tailed *t*-test; **P = 0.002). N/A, not available.

The clinical study was designed to investigate whether neurorehabilitation supported by the BSI further improves neurological recovery (Fig. 4a). Before enroling in STIMO-BSI, the participant had completed the clinical trial STIMO, which enabled him to regain volitional control over previously paralysed muscles and to improve his standing and walking functions. However, after three years of regular training with stimulation only, he had reached a plateau of recovery (Fig. 4d–f).

The participant completed 40 sessions of neurorehabilitation (Fig. 4b). which involved walking with BSI, single-joint movements with BSI, balance with BSI and standard physiotherapy. Because impairments were more pronounced in hip flexor muscles, we primarily focused the training exercises and BSI configurations on the control of these muscles.

This neurorehabilitation programme mediated pronounced improvement in the volitional control of hip flexor muscles and associated hip flexion movements without stimulation (Fig. 4c). This recovery correlated with gains in sensory (4 points in light touch sensory score) and motor scores (Fig. 4d), and enhanced standing and walking capacities that were captured in an increase in WISCI II scores from 6 before STIMO to 16 after STIMO-BSI (Fig. 4e). Concretely, the participant exhibited improvements in all the conventional clinical assessments, such as the six-minute walk test, weight-bearing capacities, timed up and go, Berg Balance Scale and walking quality assessed using the observational gait analysis scale^28^ by physiotherapists blinded to the study (Fig. 4d–h and Extended Data Table 3). These improvements without stimulation translated into a meaningful increase in quality of life, such as walking independently around the house, transiting in and out of a car or drinking a beverage with friends standing at a bar (Supplementary Video 3).

## Integration of the BSI in daily life

The BSI enhanced the standing and walking capacities of the participant, which compelled us to develop a BSI framework for independent use at home.

**Extended Data Fig. 7:**
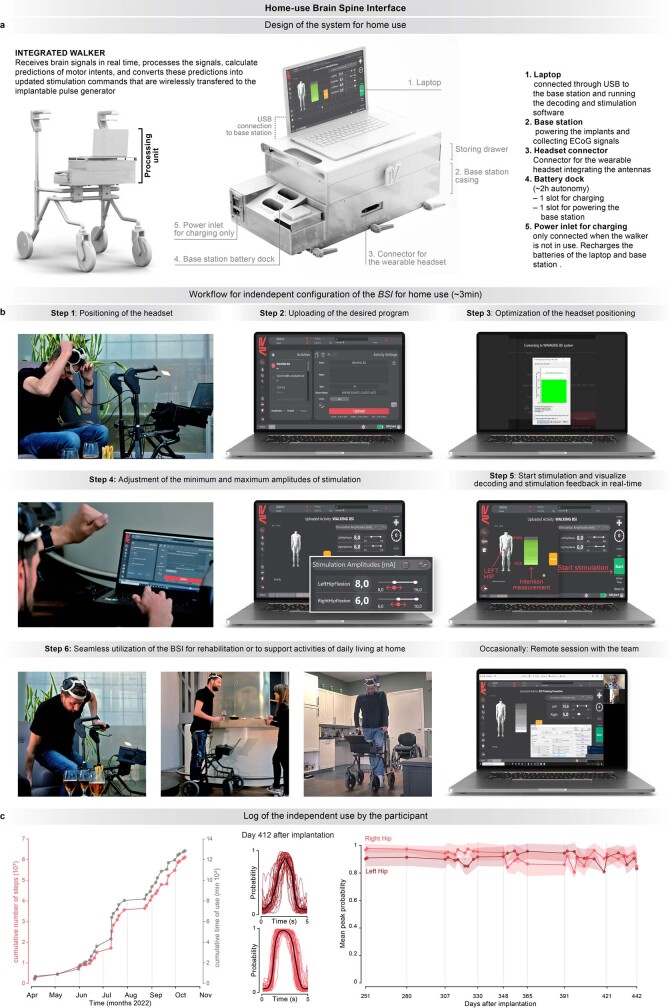
Design and configuration of the *BSI* for independent use at home. **a**, An integrated walker was designed and fabricated to incorporate the different hardware composing the *BSI*, thereby maximizing the practicability of the technological platform for use at home. The system is battery-powered and can operate autonomously for approximately 2 h without any supervision. **b**, Sequence showing the different steps to configure the *BSI*, including the positioning of the communication headset, uploading of a *BSI* program, monitoring of signal quality to ensure appropriate placement of the antennas, and adjusting the minimum and maximum amplitudes of the stimulation. The participant has been using the *BSI* independently to support neurorehabilitation and daily life activities over nearly one year. Positioning the hardware and configuring the *BSI* require approximately 5 min. **c**, Usage log and performance quantification of the participant after the main phase of the study as a cumulative number of decoded steps and cumulative time of use over a period of 181 day, i.e. since the participant returned to his home.

We designed a system that could be operated by the participant without any assistance. This system includes a walker equipped with an integrated case that embeds all the components of the BSI (Extended Data Fig. 7). A tactile-based interface enables the participant to interact with the tailored software to launch an activity, verify the placement of the headset and adjust the minimum and maximum amplitudes of stimulation programs. The configuration of the hardware and software is completed with minimal user inputs within less than 5 min, after which the participant can leverage the BSI for neurorehabilitation or to support activities of daily living (Supplementary Video 4). The participant used the system regularly over the course of 7 months with stable decoding performance (Extended Data Fig. 7c). This home use translated into a broad increase in the perceived benefits by the participant, as quantified by the Psychosocial Impact of Assistive Devices Scale (PIADS) questionnaire (Extended Data Table 4). Security, skilfulness and the ability to participate were ranked with the maximum possible gains in this questionnaire.

## Discussion

We conceived a wireless, digital bridge between the brain and spinal cord that restored natural control over lower limb movements to stand and walk on complex terrains after paralysis due to a spinal cord injury. Moreover, neurorehabilitation mediated neurological improvements that persisted even when the bridge was switched off.

The validation of this digital bridge was restricted to a single individual with severe but partial damage of the spinal cord, and it therefore remains unclear whether the BSI will be applicable to other injury locations and severities. However, several observations suggest that this approach will be applicable to a broad population of individuals with paralysis. First, the physiological principles underlying targeted epidural electrical stimulation of the spinal cord have now been validated in nine out nine treated individuals with incomplete^4^ and complete^5^ injuries^6^. Second, we developed procedures that supported straightforward, rapid and stable calibration of the link between cortical activity and stimulation programs, enabling the participant to operate the BSI at home without supervision. Third, a comparable robustness and stability of this computational and technological brain decoding framework has now been observed in two additional individuals with tetraplegia^3,26,29^. Although the previous experience of the participant with the stimulation accelerated the configuration of the BSI, we do not anticipate major impediments to implement a BSI in new individuals. Indeed, we were able to configure stimulation programs that restored stepping within one day in three participants with complete sensorimotor paralysis^5^.

The delivery of epidural electrical stimulation over the lumbar spinal cord has enabled many individuals with spinal cord injury to regain adaptive control over the activity of otherwise paralysed muscles. This recovery has been documented in various independent studies, including in participants with complete sensorimotor paralysis^5,6,30–34^. These observations indicate that anatomically intact, yet functionally silent pathways from the brain can modulate the impact of epidural electrical stimulation on the activity of the spinal cord below the injury. However, these studies also acknowledge a series of limitations. First, stimulation parameters must be fine-tuned on the basis of the targeted muscle or desired motor function. Second, the onset of the stimulation must be precisely synchronized with the motor intention. Third, finely graded control over the activity of muscles requires modulating the amplitude of the stimulation. The BSI remedies these three limitations. In this scenario, the residual and prosthetic pathways converge on the same neurons below the injury, enabling graded and sustained control over the activity of muscles. This cooperation probably plays an essential role in the reorganization of neuronal pathways that mediate neurological recovery in response to neurorehabilitation with the BSI. Comparable observations have been reported when electroencephalographic signals were coupled to an exoskeleton or functional electrical stimulation of muscles during gait rehabilitation in people with spinal cord injury^22,24,35^. However, the poor quality of electroencephalographic signals in mobile conditions, combined with the impracticality of this technological framework, are an impediment to the clinical implementation of these non-invasive strategies.

Neurorehabilitation supported by the digital bridge mediated additional neurological improvements after three years of stable performance, despite continued use of epidural electrical stimulation at home. These improvements primarily took place in the control of hip muscles, which was the main target of brain-controlled stimulation programs during neurorehabilitation. Although focused on one muscle group, this neurological recovery translated into the ability to lift the leg against gravity without stimulation. This recovery supported independent walking with crutches.

In preclinical models, neurorehabilitation supported by a digital bridge triggered superior recovery compared to epidural electrical stimulation alone^15^. Brain-controlled neuromuscular stimulation also mediated durable functional improvements of the engaged muscles after stroke^36^ and spinal cord injury^22,24,37^. As the participant had previously reached a plateau of recovery after intensive rehabilitation using spinal cord stimulation alone, it is reasonable to assume that the BSI triggered a reorganization of neuronal pathways that was responsible for the additional neurological recovery. These results suggest that establishing a continuous link between the brain and spinal cord promotes the reorganization of residual neuronal pathways that link these two regions under normal physiological conditions^38–41^. Expanding the concept of a digital bridge to the cervical spinal cord may also restore arm and hand movements after spinal cord injury^42^ and stroke^43^. However, it is important to appreciate that the relative amount of neurological recovery will necessarily correlate with the severity of the lesion.

Scaling up this digital bridge will require several developments. First, the practical utilization of the cortical implant will necessitate miniaturization of the base station, computing unit and unnoticeable antennas. Compressive sensing and dynamic adjustment of sampled electrodes and features could further reduce cortical device footprint. Second, the spinal implant must be endowed with ultrafast communication capabilities, versatile stimulation parameters and direct wireless control from the wearable computing unit. Finally, the cortical and spinal implants could be controlled by a single low-power integrated circuit embedding a neuromorphic processor with self-calibration capability that autonomously translates cortical activity into updates of stimulation programs. Although these developments require time and resources, we do not anticipate technical hurdles to realize this transition.

The concept of a digital bridge between the brain and spinal cord augurs a new era in the treatment of motor deficits due to neurological disorders.

## Methods

### Study design and participant

All experiments were carried out as part of the ongoing clinical feasibility study STIMO-BSI (‘Brain-controlled Spinal Cord Stimulation in Patients With Spinal Cord Injury’), which investigates the safety and preliminary efficacy of brain-controlled spinal cord stimulation after spinal cord injury (clinicaltrials.gov, NCT04632290). All surgical and experimental procedures were performed at the Lausanne University Hospital (CHUV) except for the magnetoencephalography experiments, which were performed at the CEA Clinatec facilities (Grenoble). The study involved functional assessments before implantation of the cortical devices, the neurosurgical procedure, a 6 week period during which various decoders were calibrated and spinal cord stimulation libraries were established, and a 15 week period of neurorehabilitation with physiotherapists. which amounted to a total of 40 sessions lasting one to three hours. The neurorehabilitation programme was personalized on the basis of the participant’s improvements. At the end of the neurorehabilitation period, the participant exited the active participation phase of the clinical trial, and was offered the opportunity to continue using the BSI at home. The participant is currently being followed up on a regular basis by the study team until the end of the three-year study extension phase of home use of the system.

Before enrolment in the STIMO-BSI study, the participant had completed the clinical protocol STIMO (‘STIMO: Epidural Electrical Simulation (EES) With Robot-assisted Rehabilitation in Patients With Spinal Cord Injury’, NCT02936453) during which a spinal cord stimulation system had been implanted and he had completed a five-month intensive neurorehabilitation programme supported by EES, followed by a two-year period of independent use at home.

Additionally, one year before joining the STIMO-BSI trial, the participant underwent a surgical procedure with: (1) talonavicular arthrodesis, transfer of the toe extensors to the peroneus tertius; and transfer of the tibialis posterior to the tibialis anterior and extensor digitorum longus; and (2) tenotomy of all long toe flexors and interphalangeal arthrodesis of the hallux. Both were performed bilaterally and could have impacted the reliability of the long toe extensor motor scores before and during the study due to change in spasticity and mechanical properties of the joint. Therefore, we decided not to report the long toe extensor motor score in our analysis.

### Pre-operative magnetoencephalography

Before entering the STIMO-BSI clinical trial, the participant was already implanted with a spinal cord system that was not MRI compatible. Therefore, we were not able to perform anatomical or functional MRI of the brain. Magnetoencephalography (MEG) is less sensitive to anatomical imprecisions for source reconstruction compared to electroencephalography (EEG)^44^. Therefore, we decided to use MEG to map the activity correlated to the limb motor intentions.

Before the neurosurgical procedure to place the cortical implants, MEG activity was measured in a magnetically shielded room using a 306-channel whole-scalp array (204 planar gradiometers and 102 magnetometers) from the Elekta Neuromag system (Elekta Neuromag). Electrocardiogram and electro-oculogram were recorded simultaneously. The recording sampling rate was 1,000 Hz. Continuous head position indicator signals were recorded during the experiments to track the head movements of the subject. Before experimentation, a three-dimensional (3D) digitization system (Isotrak II, Polhemus) was used to localize anatomical fiducial points for later coregistration with head computerized tomography (CT). Temporal signal space separation (tSSS) was applied to reduce the noise in the MEG data using MaxFilter v.3.0 software (Elekta). First, manual review of raw data enabled the marking of bad channels. Second, the tSSS filter was applied using head movement compensation and automatic bad channel correction. The main parameters were kept to default (tSSS correlation threshold of 0.98, orders of expansion for ‘in’ and ‘out’ components of signal set to 8 and 3, respectively, and a 10 s time buffer). Notch filtering at 50 Hz and harmonics (100 Hz, 150 Hz, 200 Hz and 250 Hz) were also applied to remove power line contamination. Stereotypical artefacts (cardiac, ocular) were identified by independent components analysis using MNE-Python software^45^ and rejected on visual screening (Infomax method, calculated separately for magnetometers and gradiometers using 64 components). The head, skull and cortex geometries were calculated from CT scan using the MRI segmentation routine included in the Brainstorm software^46^, followed by calculation of the head model using the overlapping spheres method. A 3D inversion kernel was calculated using Brainstorm implementation of the Minimum Norm Imaging method with default parameters. It enabled the reconstruction of cleaned raw data at brain source level for the subsequent calculations. Finally, the MSA method^47^ was used to reconstruct the brain activity related to wrist, hip and ankle motor attempts. To estimate task-specific brain activations, MSA uses cross-validated, shifted, multiple Pearson correlation, calculated from the time–frequency transformed brain signal and the binary signal of stimuli. 3D snapshots of these activations at their maximum were exported as DICOM in the original CT scan frame of reference for use in neuronavigation tools. For rendering, we manually segmented the brain from the patient pre-operative CT scan using Slicer (slicer.org) and used Blender for rendering. We recoloured the MEG signal with a red colour ramp then superposed it with the 3D render of the brain.

### Electrocorticography WIMAGINE devices

The WIMAGINE implantable recording system was designed for bilateral epidural implantation over the sensorimotor cortex^20^. Electronic components were housed in a titanium case (50 mm diameter, 7–12 mm thick and a convex external face). An array of 64 platinum–iridium (90:10) recording electrodes for epidural ECoG (2 mm in diameter, 4–4.5 mm pitch) and five reference electrodes were located on the flat inner face of the device. The ECoG data were recorded thanks to an application-specific integrated circuit^48^ that enabled multi-channel amplification and digitalization with an input referred noise of less than 0.7 μV root mean square in the 0.5 Hz–300 Hz range. Data were radio-emitted through an ultrahigh frequency antenna (402–405 MHz). Power was supplied remotely through a 13.56 MHz inductive high-frequency antenna. Both antennas were embedded in a silicone flap extending on the subcutaneous space. To ensure signal stability at high frequency (586 Hz), with regard to the limited bandwidth, 32 contacts out of the 64 were used for each implant. The wireless connection used two external antennas held in front of the recorders by a custom-designed headset. The technical specifications of the device are reported in Extended Data Table 1.

### Neurosurgical procedure

The surgery was performed under general anaesthesia. A neuronavigation station (StealthStation, Medtronic) was used to locate the centre of the craniotomies on each hemisphere. The anatomical and functional information obtained from MEG and CT scan imaging enabled the selection of the entry points to maximize the coverage of the leg region of the sensorimotor cortex, while ensuring a safe margin from the sagittal sinus area. Following a coronal incision, two circular craniotomies of 5 cm in diameter were performed using a custom-made trephine. The bone flaps were removed to expose the dura mater. The two WIMAGINE implants were placed over the dura, and then carefully suspended and secured with non-resorbable sutures. The skin was then sutured over the implants. The participant was discharged the next day. The calibration phase was initiated after a rest period of two weeks.

### Decoder architecture

ECoG data were collected from 32 channels per implant at an acquisition frequency of 586 Hz. The signals were band-pass filtered between 1 Hz and 300 Hz. The data were streamed through the fieldtrip toolbox to a custom-made decoding software running in the Matlab Runtime Environment (Mathworks).

To decode the intention to perform lower limb movements, we implemented a variant of the recursive exponentially weighted Markov-switching multilinear model (REW-MSLM) algorithm that we had previously developed to decode upper limb movements^26^. REW-MSLM is a mixture of multilinear algorithms form experts. It consists of a hidden Markov model (HMM) classifier, called ‘gate’, and a set of independent regression models, called ‘experts’. Each expert is generally dedicated to the control of a group of degrees of freedom, a specific limb or movement (for example, joint movement). The HMM-based classifier predicts the probability of such specific limb or movement activation (states) associated with a particular expert. The resulting decoder output results from soft mixing of expert predictions according to estimated probabilities.

The gate HMM-based classifier predicts the state and assumes that the state sequence *Z*(*t*) follows a first-order Markov chain hypothesis. Consequently, the probability of a state at each time step depends on the combination of the previous state and the newly acquired ECoG data. The HMM-based classifier is composed of an emission and transition probability model. At each time step, the emission probability is estimated from the observations of ECoG signals independently from the sequence of the state. In the current study, a linear discriminative classifier was used for the emission probability model. For *K* states/classes (in our case, *K* = 7 states, rest, hip, knee, ankle, bilaterally), the classifier output was computed as follows:

$${{\bf{d}}}^{{\rm{g}}{\rm{a}}{\rm{t}}{\rm{e}}}(t)={\beta }^{{\rm{g}}{\rm{a}}{\rm{t}}{\rm{e}}}X(t)+{{\bf{b}}}^{{\rm{g}}{\rm{a}}{\rm{t}}{\rm{e}}},$$

$${{\bf{d}}}^{{\rm{g}}{\rm{a}}{\rm{t}}{\rm{e}}}(t)=({d}_{1}^{{\rm{e}}{\rm{m}}{\rm{i}}{\rm{s}}{\rm{s}}{\rm{i}}{\rm{o}}{\rm{n}}}\,(t),\ldots ,{d}_{K}^{{\rm{e}}{\rm{m}}{\rm{i}}{\rm{s}}{\rm{s}}{\rm{i}}{\rm{o}}{\rm{n}}}\,(t)).$$

Here *β*^gate^ and b^gate^ are matrices of coefficients and bias of linear discriminative classifiers. Then, the emission probability vector ${\alpha }^{{\rm{e}}{\rm{m}}{\rm{i}}{\rm{s}}{\rm{s}}{\rm{i}}{\rm{o}}{\rm{n}}}\,(t)\,=$ $\left({\alpha }_{1}^{{\rm{emission}}}\,(t),\ldots ,{\alpha }_{K}^{{\rm{emission}}}\,(t)\right)$ is obtained from the classifier output d^gate^(*t*) through the softmax normalization:

$${\alpha }_{k}^{{\rm{emission}}}\,(t)=\frac{{{\rm{e}}}^{{d}_{k}^{{\rm{gate}}}(t)}}{\mathop{\sum }\limits_{i=1}^{K}{{\rm{e}}}^{{d}_{i}^{{\rm{gate}}}(t)}}.$$

Finally, the emission probabilities are weighted by the HMM state transition probabilities matrix *T*, where *T* is a *K*-by-*K* matrix with coefficients defined by the cumulative number of transitions between cued states.

$$\hat{\alpha }(t)=\frac{{\alpha }^{{\rm{e}}{\rm{m}}{\rm{i}}{\rm{s}}{\rm{s}}{\rm{i}}{\rm{o}}{\rm{n}}}\,(t)T\hat{\alpha }(t-1)}{{\alpha }^{{\rm{e}}{\rm{m}}{\rm{i}}{\rm{s}}{\rm{s}}{\rm{i}}{\rm{o}}{\rm{n}}}\,(t)T\hat{\alpha }(t-1)}.$$

The most probable state $\hat{Z}\left(t\right)$ sequence may be issued by maximizing the state probability $\hat{\alpha }(t)$ at step *t*. The state probability vector may be used to mix the decoder experts, or may be considered as one of the decoder outputs.

For experts, a multilinear regression model was used:

$${\phi }_{k}(t)={\beta }_{k}^{{\rm{e}}{\rm{x}}{\rm{p}}{\rm{e}}{\rm{r}}{\rm{t}}}X(t)+{{\bf{b}}}_{k}^{{\rm{e}}{\rm{x}}{\rm{p}}{\rm{e}}{\rm{r}}{\rm{t}}},$$

where ${\beta }_{k}^{{\rm{expert}}}$ and ${b}_{k}^{{\rm{expert}}}$ summarize coefficients of *k*th expert, *k* ∈ [1, *K*]. Finally, the mixture of expert output *U*(*t*) is computed from expert predictions *φ*_*k*_(*t*) and estimated probabilities ${\hat{\alpha }}_{k}\left(t\right)$ from the following equation: ${U}_{k}\left(t\right)={\phi }_{k}\left(t\right)\times {\hat{\alpha }}_{k}\left(t\right)\times \prod _{i\ne k}(1-{\hat{\alpha }}_{i}\left(t\right))$.

From this decoder architecture, we implemented two different control models to drive epidural spinal cord stimulation.

1. For six-joint control in static conditions, we implemented a mixture of expert predictions to enable the participant to achieve proportional control over the amplitude of stimulation. *U*(*t*) contains the analogue prediction of the relative desired amplitude of joint movement at any given time. The movement of each joint is linked to a specific stimulation protocol (electrode configuration, frequency and pulse width) defined in the library of stimulation programs (Extended Data Fig. 2b–d), whereas the predictions constituting *U*(*t*) are linearly rescaled into amplitude of stimulation (in mA) within a range of predefined values by the experimenter.
2. For the control of standing and walking in dynamic conditions, we took into consideration that these activities do not require simultaneous control over the amplitude of left and right hip flexions, because left and right steps must not occur at the same time. Therefore, we removed the layer of mixture of experts, and instead implemented a control model that drives stimulation amplitudes from the gate model output. This control model avoids the simultaneous delivery of stimulation over all the joints. Consequently, the amplitude is only modified for one joint at a time. In turn, we used the maximum estimated state probability $\max (\hat{\alpha }\left(t\right))$ to enable the participant to achieve proportional control over the amplitude of stimulation.

### Iterative online decoder calibration

REW-MSLM is a closed-loop adaptive decoder. In parallel to the current model use for predictions, the REW-MSLM decoders update their parameters on the basis of new incoming data, which enables the optimization of the parameters of the model in real time throughout the calibration session^26^. The linear emission probability model and expert models were identified using a recursive exponentially weighted N-way partial least square (REW-NPLS) algorithm. This algorithm was specifically designed for incremental and adaptive real-time multilinear decoder learning^49^. The transition matrix was identified by direct state transition counting during the calibration session. The resulting decoder was able to predict mental states as well as continuous movements.

Input features *X*(*t*) were computed from the ECoG signals and then fed to the decoder. Epochs ranging from 200 to 500 ms of ECoG signals from the 64 electrodes were created to generate a 100 ms sliding window. The epochs were mapped to the temporal frequency space with complex continuous wavelet transform (CCWT). The wavelets declined from the Morlet mother wavelet are centred around specific frequencies (2, 5:5:100, 125, 150, 200 Hz). The absolute value of the output of the CCWT was then decimated to obtain 2–5 points along the temporal modality, which defined the epoch. The prediction was computed every 100 ms. During the experiments, the REW-MSLM algorithm recursively updated the experts and gate coefficients every 15 s. The training data consist of 15 s batches of input ECoG features associated to output movement features. The output features are generated according to specific tasks given to the participant to perform motor imagery, including desired mental state for the gate update and a desired continuous movement (if any) for the expert in charge update.

When creating a model from scratch, assistance from the system can be added to the decoder output. This enables the participant to have movements already performed, even though the decoder does not predict correctly. Assistance decreases progressively as the model is calibrated, to eventually leave the participant in full control.

Model update:

Step 1: accumulate raw data and labels *d*^gate^ and *φ* over 15 s.

Step 2: compute the corresponding feature vector *X*(*t*).

Step 3: perform the partial least square regressions for the gating *d*^gate^ and experts *φ* to update the coefficients (*β*^gate^, *b*^gate^, *β*^expert^, *b*^expert^).

Step 4: update the transition matrix by adding the number of transitions. *T*(*i*,*j*) ≤ *T*(*i*,*j*) + sum((*Z*(*t* + 1),*Z*(*t*)) = (*i*,*j*)).

Prediction computation:

Step 1: compute the linear predictions of gate and experts from the current coefficients.

Step 2: apply the exponential normalization and the HMM transition step.

Step 3: mix the prediction from the gating and expert model.

### ECoG mapping procedure

To assess the spatial and spectral information in the ECoG signals to discriminate a specific task, we computed the linear regression weights associated with cued motor attempts. To map the features related to lower limb movements (hip, knee, ankle bilaterally) we recorded cortical signals during 57 (±6 s.e.m.) repetitions of each movement attempt cumulating 226 s (±25 s s.e.m.) during each state. The weights generated from this dataset were projected onto the spatial dimension for different frequency bands (0.5–10 Hz, 10–40 Hz, 40–100 Hz and 100–200 Hz) or on the spectral dimension.

### Epidural electrical stimulation

The implant to deliver epidural electrical stimulation (Extended Data Table 2) was composed of an ACTIVA RC implantable pulse generator (model 37612, Medtronic) that was interfaced with the Specify Surescan 5-6-5 paddle lead (model 977C190, Medtronic). A dedicated firmware enabled real-time uploads of stimulation tables to control electrical stimulation waveforms^5^. The patient programmer (SPTM, model 09103) was carried within a belt to align its position with the implantable pulse generator. We developed a custom-built stimulation program^5^ that sent commands to the patient programmer through a Bluetooth/infrared wireless bridge. The stimulation program enabled the definition of stimulation configurations (cathodes and anodes) and parameters (pulse width, frequencies and amplitude ranges) by an expert user^5^. This chain of software and hardware enabled real-time control of stimulation protocols with a latency inferior to 150 ms (ref. ^4^).

### Calibration of the library of stimulation programs

Electromyographic (EMG) activity was recorded bilaterally from the iliopsoas, rectus femoris, vastus lateralis, semitendinosus, tibialis anterior, medial gastrocnemius and soleus muscles with wireless bipolar surface electrodes (Delsys Trigno). Each pair of electrodes was placed over the belly of the targeted muscle, aligned longitudinally to muscle fibres. Abrasive paste (Nuprep, 4Weaver) was used for skin preparation to reduce electrode–skin resistance and improve EMG signal quality. An additional pair of surface-EMG electrodes was placed over the spine, at the thoracolumbar junction, to detect stimulation artefacts, and thus align muscle responses to the onset of stimulation. Continuous EMG signals were sampled at 2 kHz and saved to a desktop computer. EMG signals were band-pass filtered between 20 and 450 Hz. Recruitment curves were performed with pulses of increasing stimulation amplitudes, delivered every second. We implemented a grid search model to explore the different electrode configurations and frequencies to select the configurations of cathodes and anodes to achieve maximum selectivity in the recruitment of the targeted muscle groups^4^. The amplitude of muscle responses was normalized by *z*-scoring over all the configurations. For each period of stimulation, the average absolute value of *z*-score was computed. The *z*-score was then represented in a polar plot.

### Decoder calibration procedure

During the calibration of the decoders, the participant received visual cues through a custom-made interface displaying the targeted state with or without the direction of movement. The cues were generated as a pseudo-random sequence with programmable duration (2/4 s per state) or manually by the experimenter. The decoding environment enabled visualization of the duration spent in each state as well as the number of transitions between states. Once the performance of the decoder was judged sufficient by the participant and experimenter, cueing was discontinued and the participant could use the model without further calibration. From day to day, models were updated when deemed necessary. The iterative nature of the implementation facilitated these updates. Typically, the model supporting control over left hip and right hip flexions during walking was trained on the basis of 30 repetitions of each active state, whereas the resting state was predicted from 3 min of ECoG data that were acquired while the participant was performing unspecific hand and trunk movements, as well as talking to ensure robustness of the predictions.

### Decoding accuracy quantification

The accuracy of decoding predictions was quantified by computing the normalized cross-correlation between the decoded state $\hat{Z}$ and the cued state *Z* after delay compensation:

$${\rm{Decoding}}\;{\rm{accuracy}}=\frac{1}{\sum _{t}Z\left(t\right)}\sum _{t}Z\left(t-\tau \right)\hat{Z}\left(t\right),$$

where *τ* corresponds to the time at which the maximum of the cross-correlation between the cued state and the decoded state probability is reached.

### Muscle response accuracy quantification

Accuracy of muscle responses was obtained by computing the normalized cross-correlation between the decoded state $\hat{Z}$ and the thresholded EMG envelope, which was obtained with *T* = 200 ms sliding window:

$${\rm{EMG}}({\rm{t}})=\left[\frac{1}{{\rm{T}}},\underset{{\rm{t}}}{\overset{{\rm{t}}+{\rm{T}}}{\int }},|{\rm{zscore}}({\rm{emg}}(\tau ))|{\rm{d}}\tau \right] > 1$$

$${\rm{EMG}}\,{\rm{accuracy}}=1\,/\,\sum _{t}{\rm{EMG}}(t)\sum _{t}{\rm{EMG}}(t)\hat{Z}(t).$$

### Stepping accuracy quantification

When walking freely, there was no cue to quantify the decoding accuracy. To provide a quantification, we profiled the probability curves that were decoded during walking, and analysed peak value and width of the probability curves associated with left and right hip flexions. We conducted this analysis during walking at different time points, from the first session to sessions that occurred nearly one year after the neurosurgical procedure to place the cortical implant. The mean peak of probabilities, as well as the mean half-width (±s.d.) were calculated for each time point.

### ECoG spectrograms

To generate spectrograms of ECoG signals, we applied a continuous wavelet transform with a window of 500 ms and a step size of 100 ms. The difference between the averaged spectrograms from both implants was computed and normalized by applying *z*-scores over each frequency band. The colour maps of the normalized average spectrograms were scaled between −0.5 and 0.5 or between −0.5 and 1 for visualization.

### ECoG signal stability

Signal stability was assessed by quantification of the signal power during the resting state in the different frequency bands^50^. The participant was sitting in his wheelchair with eyes closed while ECoG signals were acquired over 2 min. For each session, a window of 90 s starting 20 s after the onset of recording was selected for analysis. The power spectrum density was estimated using Welch’s method. The root mean square was computed over the entire frequency band (0.5–292 Hz). The band power was measured for the following four frequency bands: 0.5–10 Hz, 10–40 Hz, 40–100 Hz and 100–200 Hz. To compensate for the different frequency bandwidths, the obtained band powers were normalized before being converted into dB. The signal-to-noise ratio was calculated for each band as the ratio of the band power versus the noise band power, which was estimated between 250–260 Hz due to the numerical filter. Root mean square, band power and signal-to-noise ratio were finally averaged across all the electrodes.

### Feature reinforcement with time

We analysed the reinforcement of cortical features linked to hip flexion attempts by computing the median spectrograms around the flexion cues in four different time periods to gather 100 events per period (−2 s to +2 s around the event). For each electrode, we computed the standard deviation of the spectrograms over all frequencies during the 4 s surrounding the events. We performed a linear fit over the 64 electrodes and four time periods.

### Walking model stability assessment

To analyse the stability of the walking models, we applied a principal component analysis (PCA) over the coefficients of each gate (idle, left hip flexion, right hip flexion) for each model. The gate vectors were composed of (64 channels × 24 frequencies) coefficients by averaging the temporal dimension. The PCA was performed over (3 gate × 44 models) samples spanning 4 months of use. Data were represented in the first three components of the PCA. We constructed an ellipsoid of 1,600 data points representing the contour curve that corresponded to a standard deviation of 1.4 for a 3D Gaussian distribution with the covariance and the mean value of each state.

### Quantitative gait analysis

EMG activity during walking was acquired bilaterally at 1,259 Hz using 16-channel wireless sensors (Delsys Trigno) placed over the iliopsoas, rectus femoris, vastus lateralis), semitendinosus, tibialis anterior and medial gastrocnemius. Kinematic recordings were acquired using a 3D motion capture system (Vicon Motion Systems). A network of 14 infrared cameras, which covered a 12 × 4 × 2.5 m^3^ workspace, was used to record the motion of markers attached to body landmarks. Data were acquired at a 100 Hz sampling rate using. A PCA was applied over a total of 26 kinematic and EMG parameters that were calculated for each gait cycle, as described previously^4^. The following parameters were included: step length, step height, knee height, knee angle and knee maximum angle, hip angle and hip maximum angle, limb angle, vastus lateralis activation, vastus lateralis stance activation, vastus lateralis swing activation, tibialis anterior activation, tibialis anterior stance activation, tibialis anterior swing activation, rectus femoris activation, rectus femoris stance activation, rectus femoris swing activation, iliopsoas activation, iliopsoas stance activation, iliopsoas swing activation, semitendinosus activation, semitendinosus stance activation, semitendinosus swing activation, medial gastrocnemius activation, medial gastrocnemius stance activation, medial gastrocnemius swing activation. Data were quantified during walking with the BSI and with closed control of stimulation based on motion sensors attached to the feet^5^. These data were compared to identical recordings obtained in healthy individuals. During overground walking with crutches, stepping attempts were detected when the knee angle dropped below 135 degrees with at least 2 s between steps. Steps were considered as failed when the step length was lower than 10 cm.

### Observational gait analysis

To analyse gait quality from video recordings, a panel of physiotherapists (*n* = 6), who were blind to experimental conditions and were not involved in STIMO or STIMO-BSI clinical trials, were asked to score different walking trials using items in a validated scoring sheet, which is described in Extended Data Table 3. This scoring sheet pooled items from the validated questionnaires G.A.I.T.^28^, SCI-FAI^51^, Tinetti Test^52^ and ref. ^53^.

### International Standards for Neurological Classification of Spinal Cord Injury

Neurological status was assessed by an experienced neurologist on the basis of the International Standards for Neurological Classification of Spinal Cord Injury (ISNCSCI), a comprehensive clinician-administered neurological examination of residual sensory and motor function quantifying spinal cord injury severity.

### Six-minute walk test

Endurance was assessed by the distance covered overground within six minutes with a standard four-wheel walker, but without any external assistance. This test was performed before and at the end of each period of neurorehabilitation of the STIMO and STIMO-BSI. Data were fitted with an exponential curve.

### Ten-metre walk test

Walking speed was assessed by a timed ten-metre walk test without any external assistance. The participant was instructed to walk with the preferred assistive device as fast as he could.

### Statistical analysis

Individual data points are represented on each figure. Measurements were taken from distinct samples, except for the observational gait analysis for which the expert physiotherapists were independently ranking the same videos. We used paired (when applicable) or unpaired one-tailed *t*-test, except as otherwise specified, with *α* = 0.05. *P* values are reported with ***P < 0.001, **P < 0.01 and *P < 0.05.

### Device explantation

Due to a subcutaneous infection to *Staphylococcus aureus* at the location of the cortical implant located on the right side, the principal investigator decided to explant the device 167 days after implantation. The second implant presented no sign of infection and remained in place, and fully functional. After recovery from the surgery and antibiotics treatment per os, neurorehabilitation and daily use could continue as planned by the protocol. Implantation of a new cortical implant was performed on 9 March 2023.

### Ethics statement

The STIMO-BSI study was approved by the Swiss authorities (Swissethics protocol number CER-VD2020-01814, Swissmedic 10000766, EUDAMED CIV-20-07-034126) and was conducted in accordance with the Declaration of Helsinki. The STIMO-BSI study is registered at ClinicalTrials.gov (NCT04632290). The STIMO study was approved by the Swiss authorities (Swissethics protocol number CER-VD PB_2016-00886, Swissmedic 10000234, EUDAMED CIV-16-02-014664), registered at ClinicalTrials.gov (NCT02936453), and was conducted in accordance with the Declaration of Helsinki. The participant signed a written informed consent before to participation. Moreover, the participant gave his consent for the material depicting himself to appear in the contribution and to be published in the journal and associated works without limit on the duration of publication, in any form or medium.

### Reporting summary

Further information on research design is available in the Nature Portfolio Reporting Summary linked to this article.

## Online content

Any methods, additional references, Nature Portfolio reporting summaries, source data, extended data, supplementary information, acknowledgements, peer review information; details of author contributions and competing interests; and statements of data and code availability are available at 10.1038/s41586-023-06094-5.

## Supplementary information

Reporting Summary

Supplementary Video 1 Design of the brain–spine interface.

Supplementary Video 2 Implementation of the brain–spine interface.

Supplementary Video 3 Neurorehabilitation and neurological recovery enabled by the brain–spine interface.

Supplementary Video 4 Independent use of the BSI by the participant.

## Figures and Tables

**Extended Data Table 1:**
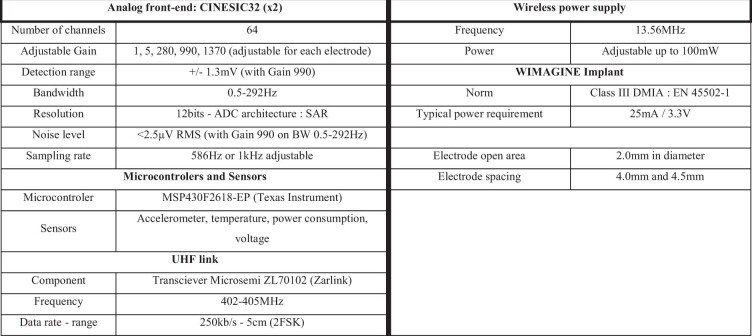
WIMAGINE device specifications

**Extended Data Table 2:**
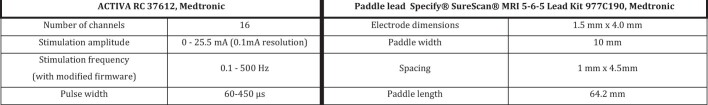
Epidural electrical stimulation system specifications

**Extended Data Table 3:**
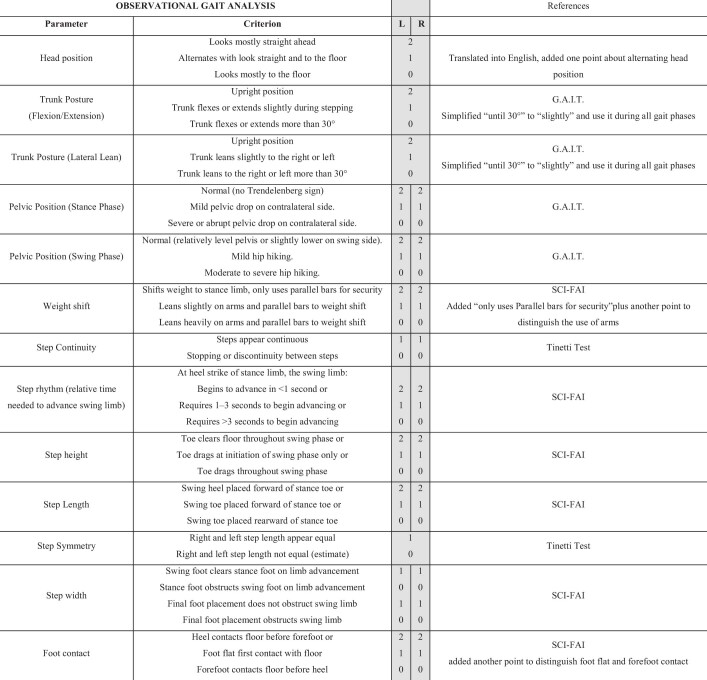
Observational gait analysis

**Extended Data Table 4:**
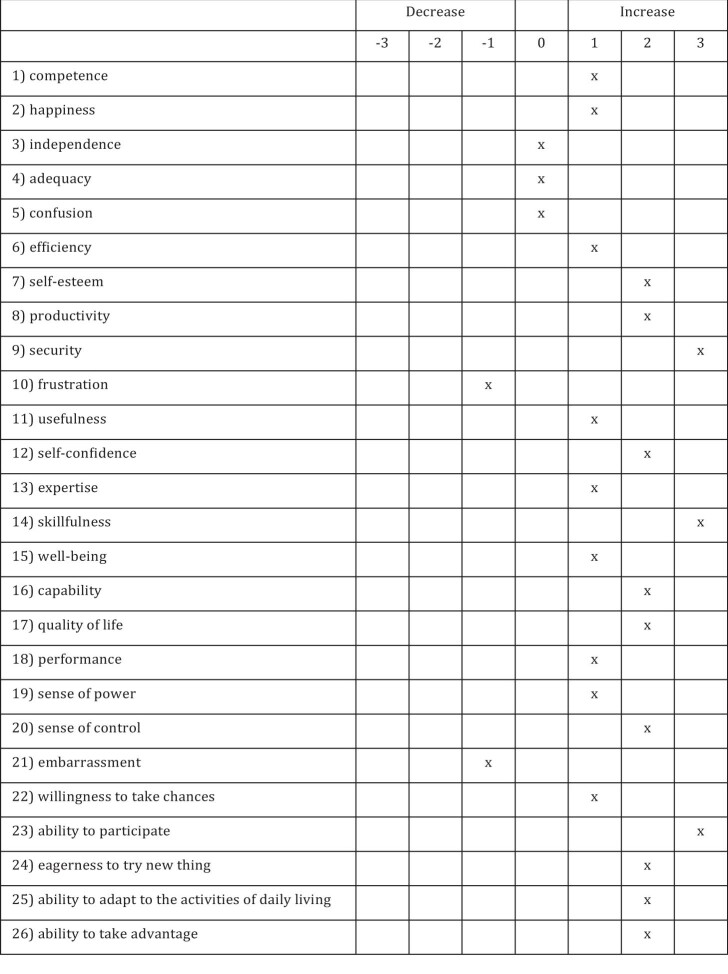
Psychosocial Impact of Assistive Devices Scale (PIADS) questionnaire during the home use phase

## Data Availability

Data presented in this manuscript are available here: 10.5281/zenodo.7680471.

## Code availability

Supporting MATLAB scripts are available here: 10.5281/zenodo.7680471.
