# Supplementary material for: Walking naturally after spinal cord injury using a brain–spine interface
Source: Nature. 2023 May 24;618(7963):126–33. doi: 10.1038/s41586-023-06094-5 (PMC10232367; doi:10.1038/s41586-023-06094-5)
Supplement: Supplementary file 1 — Reporting Summary [file 41586_2023_6094_MOESM1_ESM.pdf]

Corresponding author(s): Gregoire Courtine

Last updated by author(s): 22/02/2023

Reporting Summary

Nature Portfolio wishes to improve the reproducibility of the work that we publish. This form provides structure for consistency and transparency in reporting. For further information on Nature Portfolio policies, see our Editorial Policies and the Editorial Policy Checklist.

Statistics

For all statistical analyses, confirm that the following items are present in the figure legend, table legend, main text, or Methods section.

n/a	Confirmed	
☐	☒	The exact sample size (*n*) for each experimental group/condition, given as a discrete number and unit of measurement
☐	☒	A statement on whether measurements were taken from distinct samples or whether the same sample was measured repeatedly
☐	☒	The statistical test(s) used AND whether they are one- or two-sided<br>*Only common tests should be described solely by name; describe more complex techniques in the Methods section.*
☒	☐	A description of all covariates tested
☐	☒	A description of any assumptions or corrections, such as tests of normality and adjustment for multiple comparisons
☐	☒	A full description of the statistical parameters including central tendency (e.g. means) or other basic estimates (e.g. regression coefficient) AND variation (e.g. standard deviation) or associated estimates of uncertainty (e.g. confidence intervals)
☐	☒	For null hypothesis testing, the test statistic (e.g. *F*, *t*, *r*) with confidence intervals, effect sizes, degrees of freedom and *P* value noted<br>*Give P values as exact values whenever suitable.*
☒	☐	For Bayesian analysis, information on the choice of priors and Markov chain Monte Carlo settings
☒	☐	For hierarchical and complex designs, identification of the appropriate level for tests and full reporting of outcomes
☐	☒	Estimates of effect sizes (e.g. Cohen's *d*, Pearson's *r*), indicating how they were calculated

Our web collection on statistics for biologists contains articles on many of the points above.

Software and code

Policy information about availability of computer code

Data collection	Human Data: Magnetoencephalography data was obtained with Elekta Neuromag system (Elekta Neuromag Oy, Helsinki, Finland). Kinematic recordings were obtained at a 100-Hz sampling rate using a 3D motion capture system (Vicon Motion Systems, Oxford, UK) and the Nexus v1.8.5 software; Muscular recordings were acquired with Delsys Trigno Plugin v2.0.2 integrated in Nexus v1.8.5; Custom C# code to control stimulation in real-time in and outside the laboratory environment; Microsoft Visual Studio Community 2019 (for development in C#).
Data analysis	All softwares and software versions used to analyze data are described in the Method section at the relevant paragraph. Following is a list of softwares used: Matlab v2018a and later, Sim4Life by ZMT., Brainstorm software.

For manuscripts utilizing custom algorithms or software that are central to the research but not yet described in published literature, software must be made available to editors and reviewers. We strongly encourage code deposition in a community repository (e.g. GitHub). See the Nature Portfolio guidelines for submitting code & software for further information.

Data

Policy information about availability of data

All manuscripts must include a data availability statement. This statement should provide the following information, where applicable:

- Accession codes, unique identifiers, or web links for publicly available datasets
- A description of any restrictions on data availability
- For clinical datasets or third party data, please ensure that the statement adheres to our policy

Data that supports the findings and software routines developed for the data analysis will be made available upon reasonable request to the corresponding authors

Human research participants

Policy information about studies involving human research participants and Sex and Gender in Research.

Reporting on sex and gender

We comply with the sex and gender guidelines.

Population characteristics

One individual who had suffered a traumatic cervical SCI participated in the study. His neurological status was evaluated according to the International Standards for Neurological Classification of Spinal Cord Injury.
Key inclusion criteria:
o Having completed the main phase of the STIMO study (NCT02936453)
o Age 18-65 (women or men)
o SCI graded as American Spinal Injury Association Impairment Scale (AIS) A, B, C & D
o Level of lesion: T10 and above, based on AIS level determination by the PI, with preservation of conus function
o The intact distance between the cone and the lesion must be at least 60 mm.
o Focal spinal cord disorder caused by either trauma or epidural, subdural or intramedullary bleeding
o Minimum 12 months post-injury
o Completed in-patient rehabilitation program
o Stable medical, physical and psychological condition as considered by Investigators
o Able to understand and interact with the study team in French or English
o Adequate care-giver support and access to appropriate medical care in patient’s home community
o Agree to comply in good faith with all conditions of the study and to attend all required study training and visit
o Must provide and sign the STIMO-BSI Informed Consent prior to any study related procedures

Key exclusion criteria remain the same as for the STIMO study:
o Women who are pregnant (pregnancy test obligatory for woman of childbearing potential) or breast feeding.
o Intention to become pregnant during the course of the study,
o Lack of safe contraception, defined as: Female participants of childbearing potential, not using or not willing to continue using a medically reliable method of contraception for the entire study duration, such as oral, injectable, or implantable contraceptives, or intrauterine contraceptive devices, or who are not using any other method considered sufficiently reliable by the investigator in individual cases. Female participants who are surgically sterilised / hysterectomised or post-menopausal for longer than 2 years are not considered as being of child bearing potential.
o Other clinically significant concomitant disease states (e.g., renal failure, hepatic dysfunction, cardiovascular disease, etc.),
o Known or suspected non-compliance, drug or alcohol abuse,
o Inability to follow the procedures of the study, e.g. due to language problems, psychological disorders, dementia, etc. of the participant,
o Participation in another study with investigational drug within the 30 days preceding and during the present study,
o Previous enrolment into the current study,
o Enrolment of the investigator, his/her family members, employees and other dependent persons,
o Limitation of walking function based on accompanying (CNS) disorders (systemic malignant disorders, cardiovascular disorders restricting physical training, peripheral nerve disorders)
o History of significant autonomic dysreflexia
o Cognitive/brain damage
o Epilepsy
o Spinal canal stenosis
o Intrathecal Baclofen pump.
o Active implanted cardiac device such as pacemaker or defibrillator.
o Indication that would require diathermy.
o Indication that would require MRI.
o Increased risk for defibrillation
o Severe joint contractures disabling or restricting lower limb movements.
o Haematological disorders with increased risk for surgical interventions (increased risk of haemorrhagic events).
o Participation in another locomotor training study.
o Congenital or acquired lower limb abnormalities (affection of joints and bone).
o Spinal cord lesion due to either a neurodegenerative disease or a tumor.
o Any other anatomic or co-morbid conditions that, in the investigator’s opinion, could limit the patient’s ability to participate in the study or to comply with follow-up requirements, or impact the scientific soundness of the study results.
o Patient is unlikely to survive the protocol follow-up period of 12 months.

Recruitment	Participant recruitment was done among the previous participants of the STIMO study (NCT02936453) according to the protocol of the STIMO-BSI study (NCT04632290, clinicaltrials.gov). Previous STIMO participants were informed about the study and given the opportunity to participate to the study. The results reported here describe the first and only patient recruited as of July 2022.
Ethics oversight	This study was approved by the Swiss ethical authorities (Swissethics protocol number CER-VD2020-01814, Swissmedic protocol 10000766, EUDAMED : CIV-20-07-034126) and was conducted in accordance with the Declaration of Helsinki

Note that full information on the approval of the study protocol must also be provided in the manuscript.

Field-specific reporting

Please select the one below that is the best fit for your research. If you are not sure, read the appropriate sections before making your selection.

☒ Life sciences ☐ Behavioural & social sciences ☐ Ecological, evolutionary & environmental sciences

For a reference copy of the document with all sections, see nature.com/documents/nr-reporting-summary-flat.pdf

Life sciences study design

All studies must disclose on these points even when the disclosure is negative.

Sample size	We report the first proof-of-concept of brain-controlled spinal cord stimulation in human. Previous studies employing spinal cord stimulation or novel implanted neurotechnologies (e.g. brain machine interface) in individuals with spinal cord injury reported their results in 1 to 4 participants.
Data exclusions	None.
Replication	The sole human subject used the brain spine interface over 70 sessions including the calibration phase (30 sessions) and the rehabilitation phase (40 sessions).
Randomization	Not applicable.
Blinding	Kinematic analysis was performed by an automated pipelines. The observational gait analysis was performed by expert physiotherapists blind to the experimental condition and to the study.

Reporting for specific materials, systems and methods

We require information from authors about some types of materials, experimental systems and methods used in many studies. Here, indicate whether each material, system or method listed is relevant to your study. If you are not sure if a list item applies to your research, read the appropriate section before selecting a response.

Materials & experimental systems

n/a	Involved in the study	
☒	☐	Antibodies
☒	☐	Eukaryotic cell lines
☒	☐	Palaeontology and archaeology
☒	☐	Animals and other organisms
☐	☒	Clinical data
☒	☐	Dual use research of concern

Methods

n/a	Involved in the study	
☒	☐	ChIP-seq
☒	☐	Flow cytometry
☐	☒	MRI-based neuroimaging

Clinical data

Policy information about clinical studies

All manuscripts should comply with the ICMJE guidelines for publication of clinical research and a completed CONSORT checklist must be included with all submissions.

Clinical trial registration	NCT04632290 and NCT02936453
Study protocol	clinicaltrials.gov/ct2/show/NCT04632290 and clinicaltrials.gov/ct2/show/NCT02936453
Data collection	Data on 1 study subject was collected between July 2021 and July 2022 (STIMO-BSI) and from March 2017 to Feb 2021 (STIMO)
Outcomes	The primary endpoint is the number of device and procedure-related adverse events and device deficiencies: nature and description of adverse device effects and device deficiencies. The secondary endpoints will be the following: Functional and neuroanatomical

measures of the patient's rehabilitation stage. We will perform pre-implantation, pre-rehabilitation and post-rehabilitation procedure measures. Chosen measures: ASIA Impairment Scale, Neurobiomechanical Recordings, Gait analysis, Somato-Sensory Evoked Potential (SSEP) Measures, Voluntary Control of Muscle Contraction, Stability of the ECoG signals, BCI performance in a virtual reality environment and brain-controlled TESS condition, Modified Ashworth Scale, Berg Balance Scale, Quality of Life questionnaire: Quality of life (WHOQOL)-BREF, vital signs monitoring. Rate of adverse events not related to the device or procedure. Other Outcomes of interest include the mapping of upper limb activity. Neurobiomechanical measurements during upper limb movements will be used to isolate ECoG features that are correlated to those movements and build more efficient decoders for lower limb movements.

Magnetic resonance imaging

Experimental design

Design type: Participants were positioned supine with arms at their side.

Design specifications: Resting state structural MRI. The total scan time was <25 min overall.

Behavioral performance measures: N/A

Acquisition

Imaging type(s): Structural MRI

Field strength: 3T

Sequence & imaging parameters: 2D sagittal T2-weighted turbo spin-echo (repetition time (TR), 4560 msec; echo time (TE), 98 msec; voxel size, 0.6×0.6×3 mm3)

Area of acquisition: Thoracolumbar spine

Diffusion MRI: ☐ Used ☒ Not used

Preprocessing

Preprocessing software: *Provide detail on software version and revision number and on specific parameters (model/functions, brain extraction, segmentation, smoothing kernel size, etc.).*

Normalization: *If data were normalized/standardized, describe the approach(es): specify linear or non-linear and define image types used for transformation OR indicate that data were not normalized and explain rationale for lack of normalization.*

Normalization template: *Describe the template used for normalization/transformation, specifying subject space or group standardized space (e.g. original Talairach, MNI305, ICBM152) OR indicate that the data were not normalized.*

Noise and artifact removal: *Describe your procedure(s) for artifact and structured noise removal, specifying motion parameters, tissue signals and physiological signals (heart rate, respiration).*

Volume censoring: *Define your software and/or method and criteria for volume censoring, and state the extent of such censoring.*

Statistical modeling & inference

Model type and settings: N/A

Effect(s) tested: N/A

Specify type of analysis: ☐ Whole brain ☐ ROI-based ☐ Both

Statistic type for inference (See Eklund et al. 2016): N/A

Correction: N/A

Models & analysis

n/a	Involved in the study
☒	☐ Functional and/or effective connectivity
☒	☐ Graph analysis
☒	☐ Multivariate modeling or predictive analysis
